# Danon disease presenting with atypical non-septal sparing LGE on cardiac MRI: a case report

**DOI:** 10.1093/ehjcr/ytaf624

**Published:** 2025-12-12

**Authors:** Jiaqi Li, Lingcheng Zhu, Sen Yuan, Xin Luo

**Affiliations:** Department of Medical Imaging, Binzhou Medical University, Guanhai Road No. 346, Laishan District, Yantai, Shandong Province 264003, China; Department of Radiology, Zibo Central Hospital, No. 10, Shang Hai Street, Zibo, Shandong Province 255000, China; Department of Radiology, Zibo Central Hospital, No. 10, Shang Hai Street, Zibo, Shandong Province 255000, China; Department of Radiology, Zibo Central Hospital, No. 10, Shang Hai Street, Zibo, Shandong Province 255000, China

**Keywords:** Danon disease, Cardiovascular Magnetic Resonance, Late Gadolinium Enhancement, Case report

## Abstract

**Background:**

Danon disease is a rare genetic disorder that primarily impacts cardiac muscle, skeletal muscle, and the central nervous system. It is frequently undiagnosed in children because the characteristic cardiac symptoms are not yet apparent.

**Case summary:**

This case represents the youngest reported patient with a novel lysosome-associated membrane protein-2 variant, presenting with unexplained elevation of cardiac biomarkers, electrical abnormalities, and septal-predominant LGE on CMR, notably without left ventricular hypertrophy or dilation. Ultimately, the diagnosis of Danon disease was confirmed through genetic sequencing.

**Discussion:**

This case demonstrates that Danon disease can manifest in young patients with a specific CMR pattern—even in the absence of classic structural changes such as left ventricular hypertrophy. Therefore, clinicians should include Danon disease in the differential diagnosis for paediatric patients presenting with a combination of unexplained elevated biomarkers, electrical abnormalities, and distinctive LGE. Early genetic testing is critical in such scenarios to confirm or exclude the diagnosis.

Learning Points

- Atypical Danon disease can present without left ventricular hypertrophy or dilation.
- Danon disease can present as atypical late gadolinium enhancement (LGE) on cardiac MRI, characterized by non-septal sparing.
- Early genetic testing is critical to confirm or exclude Danon disease in the absence of classic clinical manifestations.

## Introduction

Danon disease is a rare X-linked dominant genetic disorder caused by a primary deficiency of lysosome-associated membrane protein-2 (LAMP-2).^1^ The disease typically impacts several systems, which characterized by hypertrophic cardiomyopathy, skeletal myopathy and mental retardation in male patients, and by a milder phenotype (predominantly involving cardiac muscle) in female patients.^2^ Given the complex pathogenesis of Danon disease, diagnosis generally necessitates a comprehensive approach that includes genetic testing, ultrasound, cardiac magnetic resonance (CMR), and muscle biopsies.^3^ In CMR imaging, a particular pattern of extensive late gadolinium enhancement (LGE) with mid-interventricular septum sparing may help distinguish Danon disease from other cardiomyopathies.^4^ Due to the limited understanding of Danon disease, there are presently no standardized management protocols or guidelines available.^3^

**Figure 1:**
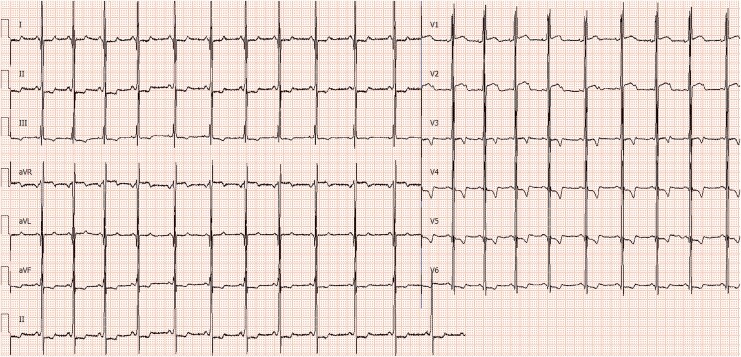
Left ventricular ECG. High voltage in the left ventricle and ST-segment elevation in the anteroseptal wall.

**Figure 2:**
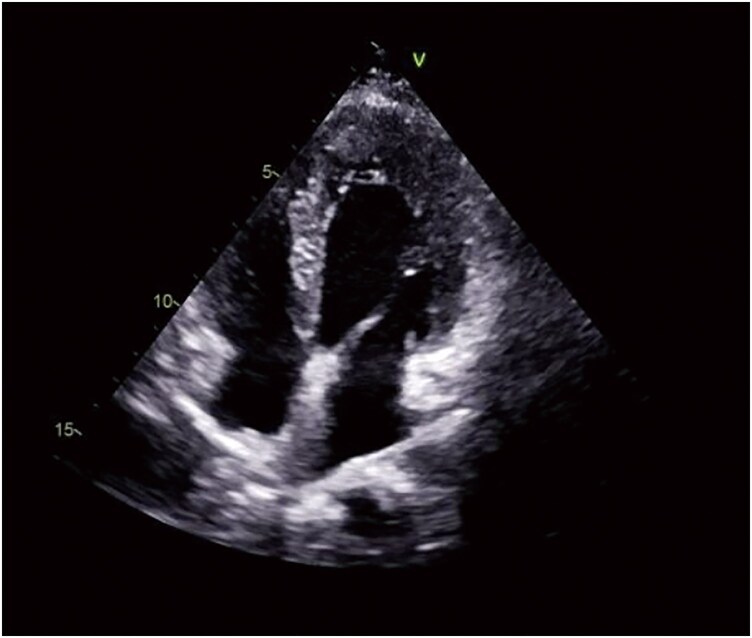
Echocardiography in the apical four-chamber view.

A 12-year-old female patient presented with a four years history of chest tightness, prompting her admission for comprehensive diagnostic and therapeutic evaluation. Her maternal history is notable for cardiomegaly. Upon admission, laboratory tests revealed elevated levels of *n*-terminal pro-B-type natriuretic peptide (NT-proBNP) at 567 pg/mL and cardiac troponin I at 38.0 ng/L, indicative of cardiac stress and injury. Cardiothoracic ratio is more than 0.5 on a routine posteroanterior chest radiograph. Subsequent cardiac assessments, including electrocardiogram and echocardiography, identified high voltage in the left ventricle and ST-segment elevation in the anteroseptal wall (*Figure 1*), with no significant thickening of the left ventricular wall (*Figure 2*).

**Figure 3:**
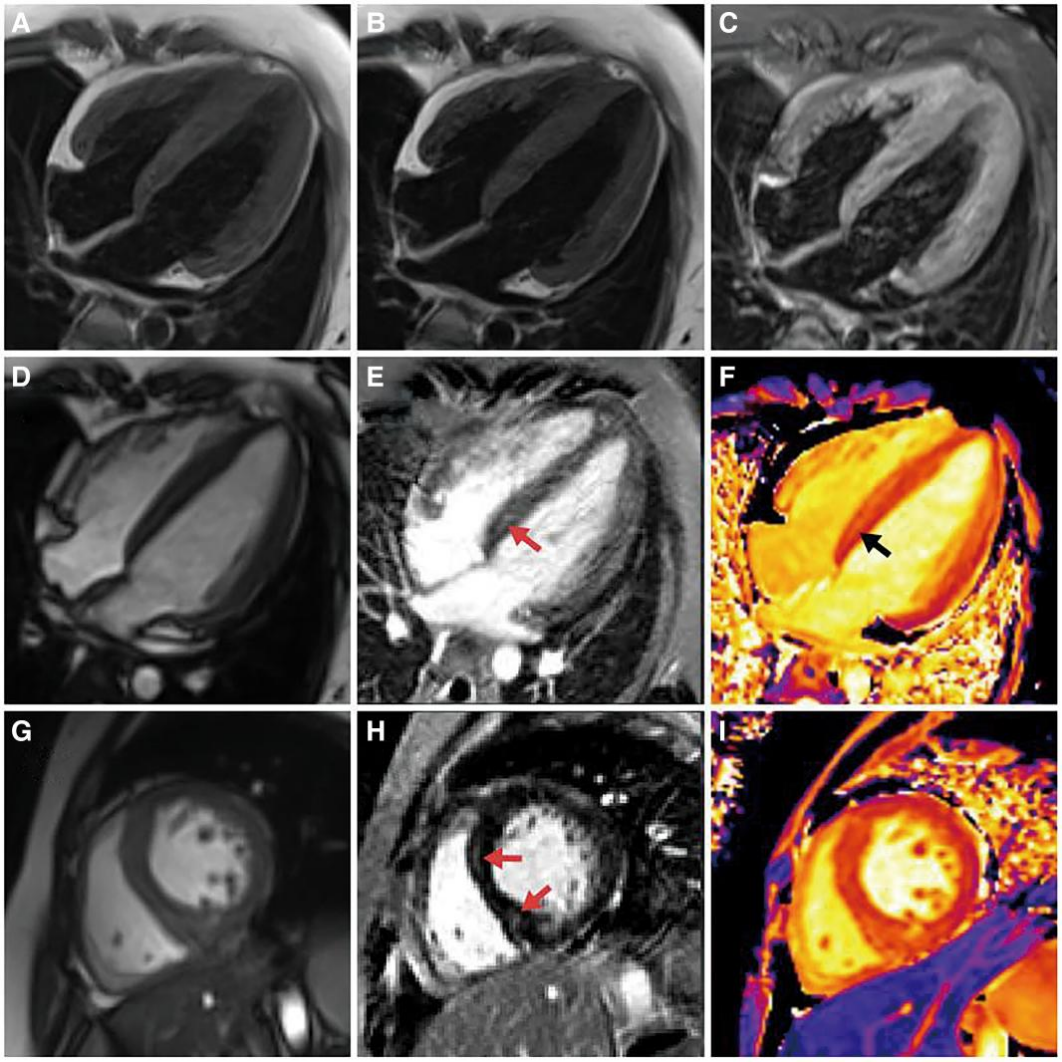
Cardiac magnetic resonance findings with basal septal mid-myocardial late gadolinium enhancement (LGE). (*A–C*) Four chamber black-blood view of T1-weighted image, T2-weighted image, T2-weighted image with fat saturation (T2FS). (*D*) Four-chamber End-diastolic SSFP cine. (*G*) Short-axis end-diastolic SSFP cine. (*E*) Four-chamber LGE image and (*H*) short-axis LGE image demonstrates a linear mid-myocardial hyperenhancement in the basal septum (arrow). (*F*) Four-chamber native T1 mapping and (*I*) short-axis native T1 mapping confirms a mildly elevated T1 value (∼1317 ms) in the basal septum (arrow).

**Figure 4:**
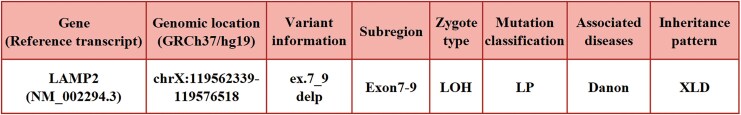
Genetic sequencing. A novel heterozygous frameshift variant in the LAMP-2 gene.

CMR imaging findings indicated that the thickest portion of the interventricular septum measured ∼8–10 mm, with a left ventricular end-diastolic diameter of 45 mm, an end-diastolic volume of 99 mL, an end-systolic volume of 29 mL, a stroke volume of 70 mL, and an ejection fraction of 70%. Linear LGE was observed in the mid-myocardial layer of the basal anterior septum (segment 2), while patchy LGE was observed in the mid-myocardial layer of the basal inferior septum (segment 3) (*Figure 3E, H*). Additionally, the native T1 values in the enhanced areas showed an increase, averaging 1317 ms (*Figure 3F, I*). The diagnosis of Danon disease was established based on the genetic sequencing, which identified a novel heterozygous frameshift variant(ex.7_9del site) in the LAMP-2 gene (*Figure 4*).

Unlike previously reported cases in the literature, this particular variant has not yet been catalogued in the HGMD database and presenting with a non-dilated, non-hypertrophied left ventricle and non-septal sparing LGE. Clinicians should consider the possibility of Danon disease when encountering these imaging features in clinical practice.

## Summary figure

**Figure:**
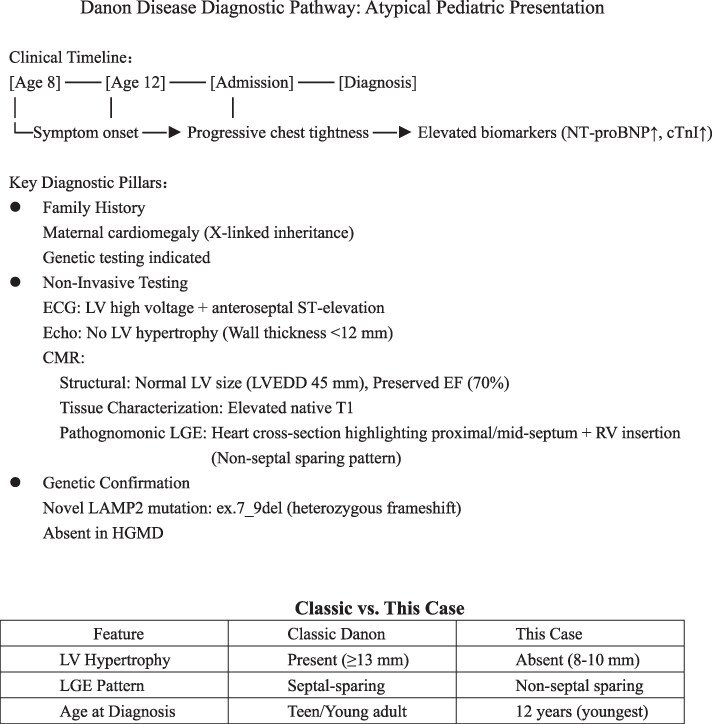


## Data Availability

No new data were generated or analysed in support of this research.

## Lead author biography

Xin Luo, a male, is the director of the Radiology Department at Zibo Central Hospital in Shandong Province and holds the position of associate chief physician. He earned his master's degree from Qingdao University Medical College, where he specialized in the imaging diagnosis of thoracic and neurological diseases. As part of an official programme, he has furthered his education at the Sourasky Medical Center in Israel.
